# Treating Heart Inflammation With Interleukin-1 Blockade in a Case of Erdheim–Chester Disease

**DOI:** 10.3389/fimmu.2018.01233

**Published:** 2018-06-01

**Authors:** Alessandro Tomelleri, Giulio Cavalli, Giacomo De Luca, Corrado Campochiaro, Teresa D’Aliberti, Moreno Tresoldi, Lorenzo Dagna

**Affiliations:** ^1^Unit of Immunology, Rheumatology, Allergy and Rare Diseases, IRCCS San Raffaele Hospital and Vita-Salute San Raffaele University, Milan, Italy; ^2^Department of Internal Medicine and Advanced Therapies, San Raffaele Hospital (IRCCS), Milan, Italy

**Keywords:** inflammation, cytokines, pericarditis, interleukin-1, anakinra, Erdheim–Chester disease

## Abstract

Pericarditis is an inflammatory heart disease, which may be idiopathic or secondary to autoimmune or auto-inflammatory diseases and often leads to severe or life-threatening complications. Colchicine and non-steroidal anti-inflammatory drugs represent the mainstay of treatment, whereas use of corticosteroids is associated with recurrence of disease flares. While effective and safe anti-inflammatory therapies remain an unmet clinical need, emerging clinical and experimental evidence points at a promising role of inhibition of the pro-inflammatory cytokine interleukin-1 (IL-1). We thus evaluated treatment with the IL-1 receptor antagonist anakinra in a case of extremely severe pericarditis with cardiac tamponade and heart failure secondary to Erdheim–Chester disease (ECD), a rare clonal disorder of macrophages characterized by rampant inflammation and multiorgan involvement. A 62-year-old man was admitted to the Emergency Department with severe pericardial effusion requiring the creation of a pleuro-pericardial window. A whole-body contrast-enhanced computed tomography pointed at a diagnosis of ECD with involvement of the heart and pericardium and of the retroperitoneal space. Over the following days, an echocardiography revealed a closure of the pleuro-pericardial window and a relapse of the pericardial effusion. Treatment with anakinra, the recombinant form of the naturally occurring IL-1 receptor antagonist, was started at a standard subcutaneous dose of 100 mg/day. After 2 days, we observed a dramatic clinical improvement, an abrupt reduction of the inflammatory markers, and a reabsorption of the pericardial effusion. Anakinra was maintained as monotherapy, and the patient remained asymptomatic in the absence of disease flares for the following year. Recent studies point at inhibition of IL-1 activity as an attractive treatment option for patients with refractory idiopathic recurrent pericarditis. Anakinra treatment may also have a role in patients with pericarditis in the setting of systemic inflammatory disorders, such as ECD.

## Background

Erdheim–Chester disease (ECD) is a rare systemic histiocytosis, characterized by infiltration of foamy macrophages into various tissues (1, 2). Infiltration of different tissues leads to the development of diverse clinical features, making diagnosing ECD a challenging task (3, 4). Common manifestations include skeletal involvement with osteosclerosis and bone pain, constitutional complaints, diabetes insipidus, neurological symptoms, pulmonary and cardiovascular involvement, and retroperitoneal infiltration, sometimes leading to ureteral obstruction (5). Involvement of the CNS or heart accounts for a very severe prognosis (6–9). The pathogenesis of ECD remains in part to be determined. ECD is currently considered an inflammatory neoplasm, characterized by oncogenic mutations along the mitogen-activated protein kinase (MAPK) pathway in macrophages, leading to rampant pro-inflammatory activation in affected tissues (10–12). In particular, activated macrophages produce high amounts of pro-inflammatory cytokines interleukin (IL)-1, IL-6, and TNF-α. Based on this understanding of ECD pathogenesis, different therapeutic approaches have been proposed for the treatment of this disease: first-line treatment with interferon (IFN)-α, treatment with small molecule kinase inhibitors, such as the BRAF inhibitor vemurafenib, and treatment with cytokine-blocking agents (13, 14).

Here, we report the case of an ECD patient with a clinical picture dominated by pericarditis with severe pericardial effusion, leading to an incumbent risk of cardiac tamponade despite a pleuro-pericardial window and prolonged treatment with colchicine and non-steroidal anti-inflammatory drugs (NSAIDs). In addition, elevated markers of systemic inflammation paralleled this organ-specific pro-inflammatory activation. Systemic and organ-specific inflammation in pericarditis is characteristically mediated by the pro-inflammatory cytokine IL-1, as pointed out by sound clinical and experimental evidence (15–17). Unequivocal confirmation to this critical role of IL-1 in pericardial inflammation comes from clinical experience with pharmacological inhibitors of this cytokine. Anakinra, the recombinant form of the IL-1 receptor antagonist that effectively blocks the activity of IL-1 (18–21), revealed unprecedented efficacy in the treatment of idiopathic recurrent pericarditis (IRP) refractory to conventional therapies (17, 22, 23). However, there are no reports to date of the use of anakinra for the treatment of pericarditis in the setting of ECD.

## Case Report

A 62-year-old man was admitted to the Emergency Department for dyspnea and chest pain. His medical record was notable for unilateral renal agenesis. He worked as a clerk and did not smoke, drink alcohol, or use illicit drugs. He had neither a family nor a personal history of cancer or autoimmune diseases; however, he reported having been hospitalized twice during the previous year for recurrent pericardial effusions, for which he had been diagnosed with IRP and was receiving treatment with colchicine and NSAIDs.

On examination, blood pressure was 120/60 mmHg, heart rate 96 beats per minute, and respiratory rate 30 breaths per minute, with normal oxygen saturation. Physical examination revealed muffled heart sounds, but no jugular vein distention or paradox pulse. Blood tests revealed mild anemia, mildly elevated troponin levels (43 ng/L, normal values < 14), elevated levels of pro-BNP (3,600 pg/mL, normal values < 227), and strikingly high levels of inflammatory markers C-reactive protein (CRP, 135 mg/L, normal values < 6), erythrocyte sedimentation rate (ESR, 118 mm/1 h, normal values < 20), and ferritin (640 ng/mL, normal values 15–150 ng/mL). A full autoantibody panel, including rheumatoid factor, anti-citrullinated protein antibody, antinuclear antibodies, cryoglobulins, and anti-neutrophil cytoplasmic antibodies, was negative. Electrocardiogram showed diffuse ST-segment elevation. Trans-thoracic echocardiography revealed ubiquitous pericardial effusion, with a maximum thickness of 45 mm. Following ineffective attempts to drain the excess pericardial fluid, a pleuro-pericardial window was surgically created. Pericardial fluid analysis revealed elevated concentrations of lactate dehydrogenase and total protein. A search for cancer cells and *Mycobacterium tuberculosis* through polymerase chain reaction was negative, as were Gram stain and bacterial culture studies.

**Figure 1:**
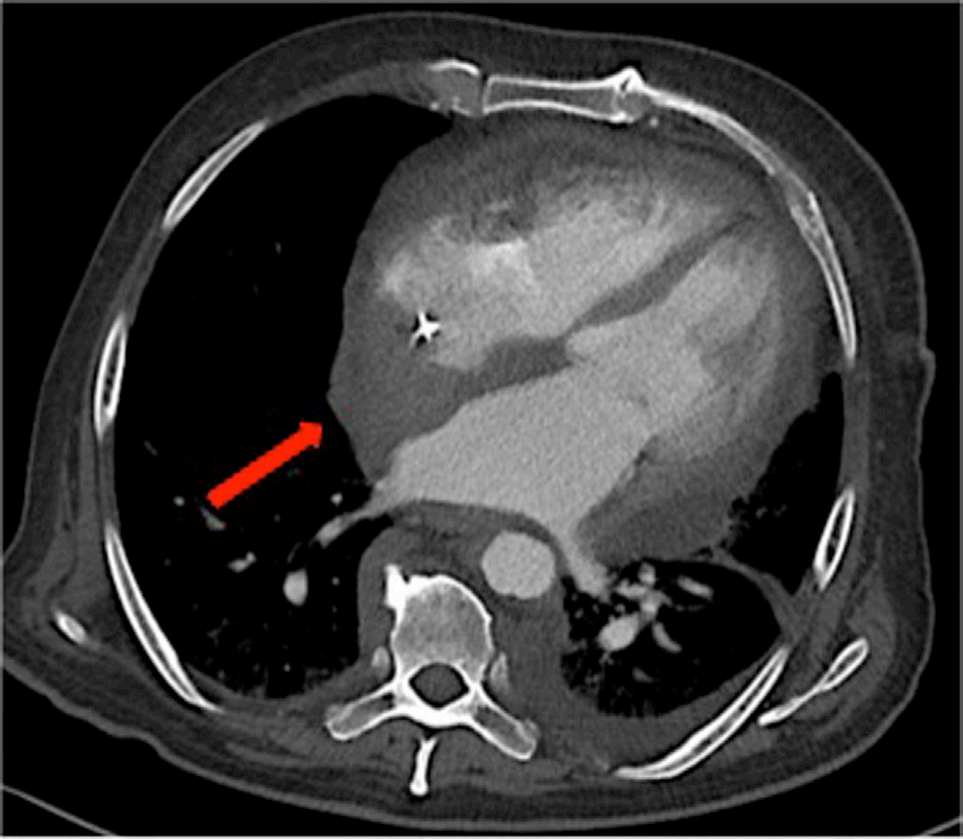
A contrast-enhanced computed tomography showing thickening of the pericardial sheets, pericardial effusion and a pseudo-tumoral infiltration of the right atrium [*red arrow*].

**Figure 2:**
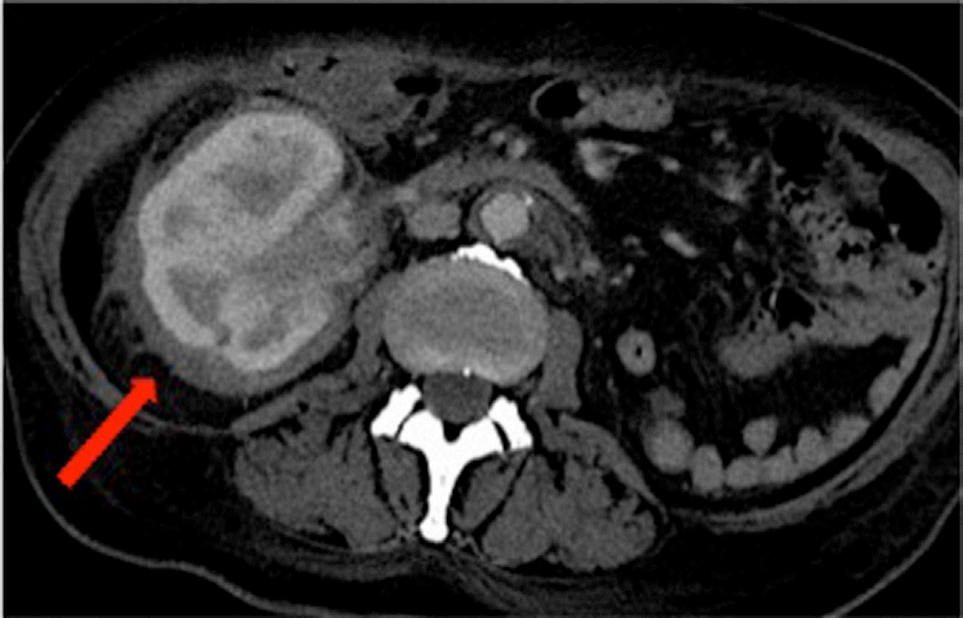
Computed tomography scan of the abdomen revealing solid tissue surrounding the right kidney (commonly referred to as “hairy kidney”) [*red arrow*] and circumferential peri-aortic sheathing (“coated aorta”).

**Figure 3:**
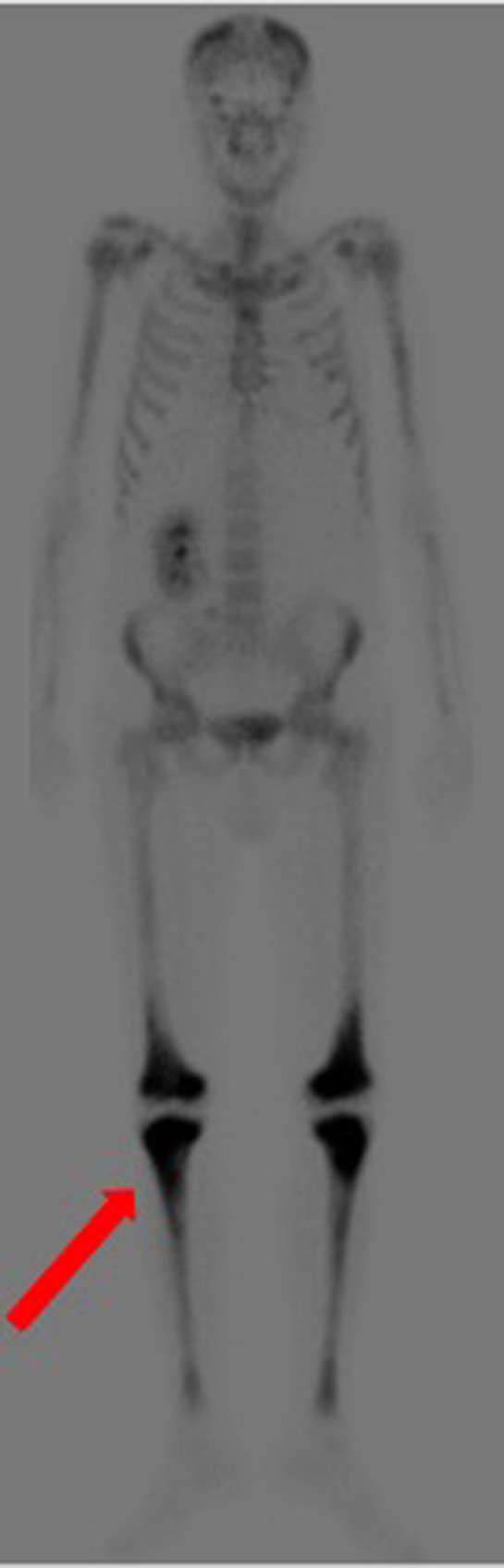
Bone scan demonstrating symmetric diametaphyseal radiotracer uptake in the long bones of the lower limbs [*red arrow*].

Over the following days of hospitalization, dyspnea and chest pain progressively worsened. Repeated echocardiography revealed a closure of the pleuro-pericardial window and relapse of the pericardial effusion, with a maximum thickness of 40 mm. A whole-body contrast-enhanced computed tomography (CT) confirmed pericardial effusion with thickening of the pericardial sheets, while also revealing a pseudo-tumoral infiltration of the right atrium (Figure 1). Moreover, abdominal scans revealed solid tissue surrounding the right kidney and aorta (Figure 2). Together with cardiac involvement, these findings of retroperitoneal involvement with peculiar “coated aorta” and “hairy kidney” radiologic signs pointed at a diagnosis of ECD. Since the skeletal system is almost invariably involved in ECD, a bone scan was then requested, which revealed pathognomonic symmetric radiotracer uptake in the long bones of the lower limbs (Figure 3). A CT-guided biopsy of the perinephric solid tissue was performed. Histology studies identified xanthogranulomatous infiltration with foamy histiocytes staining positive for CD68, but negative for S100 and for CD1a, all findings diagnostic for ECD. Molecular techniques revealed the presence of the BRAFV600E mutation in pathological histiocytes, as described (24).

Once a diagnosis of ECD was established, a critical problem to be faced was which pharmacological approach to choose following failure of the pleuro-pericardial window. The traditional drug of choice in ECD is IFN-α (7). However, we preferred not to choose IFN-α, as previous experience suggests limited efficacy in cases of severe heart inflammation (25, 26). As a valid alternative, we focused on vemurafenib, a small molecule inhibitor of BRAFV600E, which is highly effective in the treatment of ECD (27). However, efficacy of vemurafenib is normally observed only after months of treatment (7, 27), while the pericardial effusion of our patient was quickly worsening and dictating the need for a rapid intervention. The last alternative therapeutic strategy involved the use of anti-cytokine agents. The main anti-cytokine therapies that have been investigated in ECD are (1) anakinra, the recombinant form of the naturally occurring IL-1 receptor antagonist, (2) infliximab, a TNF-α monoclonal chimeric antibody, and (3) tocilizumab, a monoclonal antibody blocking the IL-6 receptor (13, 28, 29). Between these three drugs we chose anakinra, because of its rapid onset of action, excellent safety profile, and—above all—the demonstrated efficacy in the treatment of idiopathic pericarditis, as well as ECD (30, 31).

Anakinra was thereby started at a standard daily subcutaneous dose of 100 mg (20). Already 2 days after initiation of anakinra treatment, laboratory tests showed a steep reduction of ESR and CRP levels (22 mm/1 h and 16 mg/L, respectively), and the patient reported a marked improvement of fatigue and asthenia. In the following days, repeated echocardiography evaluations revealed progressive reabsorption of the pericardial effusion. The patient was thereby discharged and re-evaluated after 2 months of daily anakinra treatment. At this point, inflammatory markers had completely normalized, hemoglobin levels increased, and the patient reported no signs of cardiac failure and a considerable improvement in quality of life. A new echocardiography showed a substantial reduction of pericardial effusion, which had a maximum thickness of 16 mm. Anakinra was maintained as monotherapy, and the patient remained asymptomatic in the absence of disease flares for the following 1 year. Imaging restaging of ECD confirmed improvement of heart involvement and stability of retroperitoneal fibrosis.

## Discussion

This study reports the dramatic efficacy of IL-1 blockade in a rare case of ECD causing life-threatening pericarditis. This patient had endured repeated episodes of pericarditis requiring hospitalization despite treatment with colchicine and NSAIDs and was eventually diagnosed as having ECD, a rare inflammatory disorder characterized by involvement of various tissues, including the heart and pericardium. The latest relapse of pericardial inflammation led to severe pericardial effusion. Attempts to create a pleuro-pericardial window were unsuccessful, and pericardial effusion was rapidly increasing, leading to incumbent risk of cardiac tamponade. Treatment with IL-1 blockade was initiated given the pivotal role of IL-1 in the pathogenesis of heart and pericardial inflammation in general, as well as ECD. In addition, previous experience with IL-1 blockade in the treatment of life-threatening heart inflammation revealed an extremely rapid onset of action, which was desperately needed in this patient (17, 22).

ECD is a clonal, inflammatory disease of macrophages, which infiltrate multiple tissues leading to protean clinical manifestations. ECD is characterized by activating somatic mutations along the MAPK or related pathways in affected macrophages, leading to production of pro-inflammatory cytokines and local and systemic inflammatory activation (10, 32). Cardiovascular involvement is a frequently overlooked complication of ECD and associates with poor prognosis (33). Frequent findings are pseudo-tumoral heart infiltration, perivascular circumferential thickening of the aorta (“coated aorta”), and pericardial involvement. Pericardial involvement may rarely lead to severe pericardial effusion and hemodynamic compromise (33). To the best of our knowledge, in all cases of symptomatic pericardial effusion due to ECD described in the medical literature, a first-line pharmacological approach with IFN-α has proven ineffective. In some cases, a surgical procedure such as pleural–pericardial window or pericardiectomy was needed; other patients developed cardiac tamponade despite IFN-α treatment and did not survive (25, 26). The pericardial fluid from ECD patients with pericardial involvement is highly inflammatory, with increased levels of pro-inflammatory cytokines (14, 28). It is thus not unexpected that biological agents specifically inhibiting pro-inflammatory molecules might be effective in the treatment of ECD heart involvement. Anakinra is an established treatment option for ECD (7, 34); a previous study reported its efficacy in a case of right atrium heart wall infiltration causing rhythm disturbances (35). However, prior to this report, there was no evidence of the effectiveness of anakinra in the setting of ECD-related pericarditis and pericardial effusion.

Besides a role in the pathogenesis of inflammation associated with ECD, IL-1 is critical to the pathogenesis of pericardial inflammation in general. IL-1 mediates pericarditis, an inflammatory disease of the pericardial sheets clinically manifested with chest pain, dyspnea, and pericardial effusion. Pericarditis is traditionally included in the spectrum of auto-inflammatory conditions, characterized by flares of seemingly unprovoked, rampant inflammation typically mediated by IL-1 (36, 37). Several safety mechanisms and regulatory molecules prevent unwanted activation of IL-1 (32, 38, 39). In addition, IL-1β is produced by monocytes–macrophages as an inactive precursor and requires processing by an intracellular molecular complex (the “inflammasome”) for activation (40). In some auto-inflammatory diseases, causative mutations resulting in deregulated activation of the inflammasome and release of active IL-1 have been identified (41).

In the majority of cases of pericarditis, no specific cause can be documented, so the disease is defined as “idiopathic recurrent pericarditis.” However, pericarditis may also occur in patients affected by systemic inflammatory diseases, such as ECD. At present, NSAIDs and colchicine represent the mainstay of treatment of pericarditis, while corticosteroids are associated with increased risk of relapse (30, 42). Nevertheless, these strategies are not effective in a sizeable proportion of patients, who experience a chronic disease course marked by frequent relapses. Given the central role of IL-1 in the pathogenesis, it is not surprising that selective pharmacological blockade of this cytokine demonstrated dramatic efficacy in the treatment of pericarditis (30). The first long-term cohort study was published in 2014 and described the efficacy of anakinra in 10 patients affected by IRP, (31). Subsequent studies analyzed 34 patients with IRP, either resistant or unable to tolerate conventional therapies, who also exhibited a response to anakinra (23). Strikingly, all cases described promptly achieved disease control, within 1 week of treatment.

Given the rapidly worsening clinical picture and the potential for progression to a life-threatening situation, IL-1 blockade with anakinra was our choice, due to its extremely rapid onset of action (20). The established efficacy of anakinra in the treatment of IRP substantiated our decision, and anakinra proved highly effective in the treatment of this patient. Of note, anakinra has a remarkable record of safety. As for all biological agents, there is an increased risk of infections, but these are mostly upper airway viral rather than opportunistic infection (20). An optimal safety profile is also afforded by the short half-life of anakinra (6 h), which allows for prompt discontinuation. The most frequent side effect of anakinra, which was not experienced by our patient, is represented by injection site reactions. These can be particularly uncomfortable due to the need for daily subcutaneous administrations, but usually resolve within 2–3 weeks of treatment initiation.

## Conclusive Remarks

Regardless of the causative condition, ranging from idiopathic to rare diseases such as ECD, the inflammatory response in pericarditis escalates to an auto-inflammatory cycle leading to rampant inflammation and pericardial effusion. Prompt pharmacological inhibition of IL-1 can arrest the progression of uncontrolled inflammation, thus preventing damage and restoring homeostasis. Given the dual efficacy against pericardial inflammation and ECD, IL-1 inhibition represents a particularly suitable treatment option for ECD patients with heart involvement. Of note, IL-1 receptor blockade with anakinra is characterized by an extremely rapid onset of action and an excellent safety profile, thus being suitable for the treatment of critical, life-threatening conditions. Confirmatory studies of IL-1 inhibition in ECD heart involvement are warranted.

## Ethics Statement

The patient gave his written consent to treatment administration and to the publication of the present report.

## Author Contributions

All authors contributed substantially to this work, have approved the manuscript, and agreed with its submission.

## Conflict of Interest Statement

The authors declare that the research was conducted in the absence of any commercial or financial relationships that could be construed as a potential conflict of interest.
